# Fabry Disease in a Patient With Thin Basement Membrane Nephropathy

**DOI:** 10.7759/cureus.81876

**Published:** 2025-04-08

**Authors:** Eugene K Yeboah, Surya V Seshan, FNU Veerban, Mir Sulayman Khan, Subodh Saggi

**Affiliations:** 1 Internal Medicine, State University of New York Downstate Medical Center, Brooklyn, USA; 2 Pathology and Laboratory Medicine, Weill Cornell Medicine, New York, USA; 3 Nephrology, State University of New York Downstate Medical Center, Brooklyn, USA

**Keywords:** alpha-galactosidase a deficiency, fabry disease, lipid storage disease, thin basement membrane nephropathy, zebra bodies

## Abstract

Diagnosing Fabry disease can be challenging due to its broad spectrum of clinical presentations, highlighting the need for a high index of suspicion. A 59-year-old male patient with personal and family history of persistent hematuria was referred to our renal clinic for workup. Genetic workup confirmed a heterozygous inheritance pattern of alpha-4 chain of type IV collagen. A renal biopsy revealed characteristic electron microscopic findings typical of a lipid storage disease within the glomerular podocytes, reminiscent of Fabry disease. This case in an older adult patient underscores the variability of clinical presentation in Fabry disease, together with another inherited type IV collagen disease and the critical role of renal biopsy in diagnosing atypical cases.

## Introduction

Fabry disease was first described independently by a dermatologist, Dr Johannes Fabry, and a surgeon, Dr William Anderson, in 1898 [1]. It is the second most common lipid storage disorder in humans, after Gaucher disease [2]. The prevalence of classic Fabry disease is estimated to range from 1:8,454 to 1:117,000 males [3,4]. It is seen across all ethnic and racial groups [5]. It has a variable clinical presentation and often remains undiagnosed due to its overlap with other conditions [6]. This variability of presentation emphasizes the importance of awareness of the condition and making an accurate diagnosis [6]. Studies on kidneys used for kidney transplantation suggest that the frequency of thin glomerular basement membrane nephropathy (TBMN) in the general population may be as high as 5-9% [7,8]. One-half to two-thirds of patients with TBMN have an identifiable family history, typically inherited in an autosomal dominant pattern [9].

Fabry disease is diagnosed when there is a deficiency or gene mutation of alpha-galactosidase. However, in some instances, confirmation requires histopathological evaluation of the affected organ. Our patient was initially suspected of having thin glomerular basement membranes belonging to the Alport spectrum of disease, confirmed by the detection of a heterozygous inheritance pattern of alpha-4 chain of type IV collagen. However, his biopsy also revealed features of lysosomal lipid storage disease within the podocytes resembling Fabry disease. This underscores the critical role of histopathology in diagnosing two diseases that are potentially associated with genetic mutations, particularly when they have atypical presentations, where targeted organ evaluation is essential.

## Case presentation

A 59-year-old male patient with a history of hypertension for more than 15 years and stage 2 chronic kidney disease was referred to our renal clinic in 2015 for workup of gross hematuria. The patient described hematuria as mild to moderate, gradual in onset, intermittent, occurring at any time of the day, and not associated with fever, night sweats, weight loss, or pedal swelling. The patient had no history of sudden rise in blood pressure, recent infection, trauma, use of anticoagulants, urinary tract infections, stones, malignancy, non-steroidal anti-inflammatory drug use, joint pain, alopecia, skin rashes, oral ulcers, diarrhea, or any other complaint. However, the patient’s family history was remarkable for hematuria. The patient’s sister and son both had gross hematuria. The patient occasionally drank alcohol but did not smoke or use any illicit drugs.

The patient’s physical examination was notable for a heart rate of 97 beats/minute, respiratory rate of 16 breaths/minute, saturation on room air of 100%, blood pressure of 137/89 mmHg, and BMI of 27.9 kg/m2. The patient was afebrile, euvolemic, not jaundiced, not pale, and had no pedal swelling. The patient’s chest was clinically clear, and the abdominal examination was unremarkable. He was conscious, alert, oriented in time, place, and person. Initial Laboratory work done in 2015 is summarized in Table 1.

**Table 1:** Initial laboratory workup done in 2015. HPLC: high-performance liquid chromatography

Parameter	Patient values	Reference range
Comprehensive metabolic panel		
Sodium	139 mmol/L	136–145 mmol/L
Potassium	4.0 mmol/L	3.5–5.1 mmol/L
Calcium	9.4 mg/dL	8.2–10.0 mg/dL
Chloride	105 mmol/L	98–107 mmol/L
Creatinine	0.84 mg/dL	0.7–1.3 mg/dL
Blood urea nitrogen	16 mg/dL	7–25 mg/dL
Carbon dioxide	29 mmol/L	21–31 mmol/L
Random blood glucose	85 mg/dL	70–99 mg/dL
Creatine kinase	112 μ/L	30–223 μ/L
Estimated glomerular filtration rate	110 mL/minute/1.73m²	>60 mL/minute/1.73m²
Liver function test		
Total bilirubin	0.3 mg/dL	0.3–1.0 mg/dL
Albumin	4.39 g/dL	3.5–5.7 g/dL
Total protein	7.1 g/dL	6.0–8.3 g/dL
Aspartate aminotransferase	18 U/L	13–39 U/L
Alanine aminotransferase	18 U/L	7–52 U/L
Alkaline phosphatase	83 U/L	34–104 U/L
Glycated hemoglobin	5.4%	<5.7%
Complete blood count		
Hemoglobin	13.2 g/dL	14.0–18.0 g/dL
White blood count	6.92 k/μL	3.5–10.8 k/μL
Platelet	210 k/μL	130–400 k/μL
Hematocrit	39.2%	42.0–52.0%
Urinalysis		
Appearance	Clear	Clear
pH	5.0	5.0–8.0
Specific gravity	1.015	1.005–1.030
Urine glucose	Negative	Negative
Urine blood	Positive	Negative
Urine leucocyte esterase	Negative	Negative
Urine protein	Negative	Negative
Urine nitrite	Negative	Negative
Urine urobilinogen	<2	<2
White blood cells (urine)	<1/hpf	0–5/hpf
Urine cast	21/hpf	0–2/hpf
Random urine protein	14 mg/dL	<12 mg/dL
Glomerulopathy workup		
Complement (C3) levels	141.3 mg/dL	83–200 mg/dL
Complement (C4) levels	30.2 mg/dL	16–47 mg/dL
Complement total (CH50)	58 U/mL	42–62 U/mL
Antistreptolysin O antibodies	26.2 IU/mL	0.0–200.0 IU/mL
Immunoglobulin A serum	484.3 mg/dL	69.0–378.0 mg/dL
Immunoglobulin G serum	1,160.8 mg/dL	694.0–1,618.0 mg/dL
Immunoglobulin M serum	39.5 mg/dL	60.0–263.0 mg/dL
Serum-free kappa light chains	13.73 mg/dL	3.30–19.40 mg/dL
Serum lambda light chains	13.59 mg/dL	5.71–26.30 mg/dL
Free kappa/lambda ratio	1.01	0.26–1.65
Infectious workup		
Human immunodeficiency virus 1/2 antigen/antibodies	Negative	Negative
Hepatitis C	Non-reactive	Non-reactive
Hepatitis B surface antigen	Non-reactive	Non-reactive
Tuberculosis QuantiFERON Gold	Negative	Negative
Serum rapid plasma reagin	Negative	Negative
Autoimmune workup		
Antinuclear antibody	Negative	Negative
Anti-double-stranded DNA	<1 IU/mL	<29 IU/mL
Aldolase	5.5 U/L	3.3–10.3 U/L
Glomerular basement membrane antibodies	3 U	0–20 U
Proteinase-3 antibodies	<3.5 U/mL	0.0–3.5U/mL
Myeloperoxidase antibodies	<9.0 U/mL	0.0–9.0 U/mL
Hemoglobin electrophoresis HPLC		
Hemoglobin A	97.2%	93.0–98.0%
Hemoglobin A2	2.6%	1.5–3.6%
Hemoglobin F	0.2%	0.0–2.5%

The patient was referred to a urologist for workup of hematuria, where both urine cytology and cystoscopy were negative for malignancy. His cystoscopy showed moderate bladder trabeculations but no evident bladder pathology. Renal biopsy was recommended but deferred, following which he was lost to follow-up. He returned to us in 2024 with recurrent symptoms and finally agreed to all workups, including a kidney biopsy. Laboratory work done in 2024 is summarized in Table 2.

**Table 2:** Laboratory workup done in 2024.

Parameter	Patient values	Reference range
Comprehensive metabolic panel		
Sodium	140 mmol/L	136–145 mmol/L
Potassium	4.2 mmol/L	3.5–5.1 mmol/L
Calcium	9.0 mg/dL	8.2–10.0 mg/dL
Chloride	102 mmol/L	98–107 mmol/L
Creatinine	0.7 mg/dL	0.7–1.3 mg/dL
Blood urea nitrogen	13 mg/dL	7–25 mg/dL
Carbon dioxide	28 mmol/L	21–31 mmol/L
Random blood glucose	79 mg/dL	70–99 mg/dL
Estimated glomerular filtration rate	110 mL/minute/1.73m²	>60 mL/minute/1.73m²
Liver function test		
Total bilirubin	0.3 mg/dL	0.3–1.0 mg/dL
Albumin	4.2 g/dL	3.5–5.7 g/dL
Total protein	7.0 g/dL	6.0–8.3 g/dL
Aspartate aminotransferase	16 U/L	13–39 U/L
Alanine aminotransferase	14 U/L	7–52 U/L
Alkaline phosphatase	87 U/L	34–104 U/L
Complete blood count		
Hemoglobin	13.0 g/dL	14.0–18.0 g/dL
White blood count	6.35 k/μL	3.5–10.8 k/μL
Platelet	202 k/μL	130–400 k/μL
Urinalysis		
Appearance	Clear	Clear
pH	6.0	5.0–8.0
Specific gravity	1.008	1.005–1.030
Urine glucose	Positive	Negative
Urine blood	Positive	Negative
Urine leucocyte esterase	Negative	Negative
Urine protein	Negative	Negative
Urine nitrite	Negative	Negative
Urine urobilinogen	<2	<2
White blood cells (Urine)	<1/hpf	0-5/hpf
Urine cast	21/Ipf	0-2/Ipf
Random urine protein	7 mg/dL	<12 mg/dL
Coagulation		
Prothrombin time	11.2 seconds	10.8–13.7 seconds
Activated partial thromboplastin time	33.3 seconds	25–35 seconds
International normalized ratio	0.9	<1
Infectious workup		
Human immunodeficiency virus antigen/antibodies	Negative	Negative
Hepatitis C	Non-reactive	Non-reactive
Hepatitis B surface antigen	Non-reactive	Non-reactive
Tuberculosis QuantiFERON Gold	Negative	Negative
Serum rapid plasma reagin	Negative	Negative
Genetic testing		
Alpha-galactosidase S	0.228 U/L	0.074-0.457 U/L
Pathologic variant of the alpha-4 chain of type IV collagen	Positive	Negative

The patient’s spot urine protein and urine creatinine were 7 mg/dL and 29 mg/dL, respectively; hence, his spot urine protein to creatinine ratio (UPCR) in 2024 was 241 mg/g (normal UPCR is <150 mg/g). Hence, the patient had moderate proteinuria. This was the only positive finding in his initial laboratory workup.

Imaging

**Figure 1:**
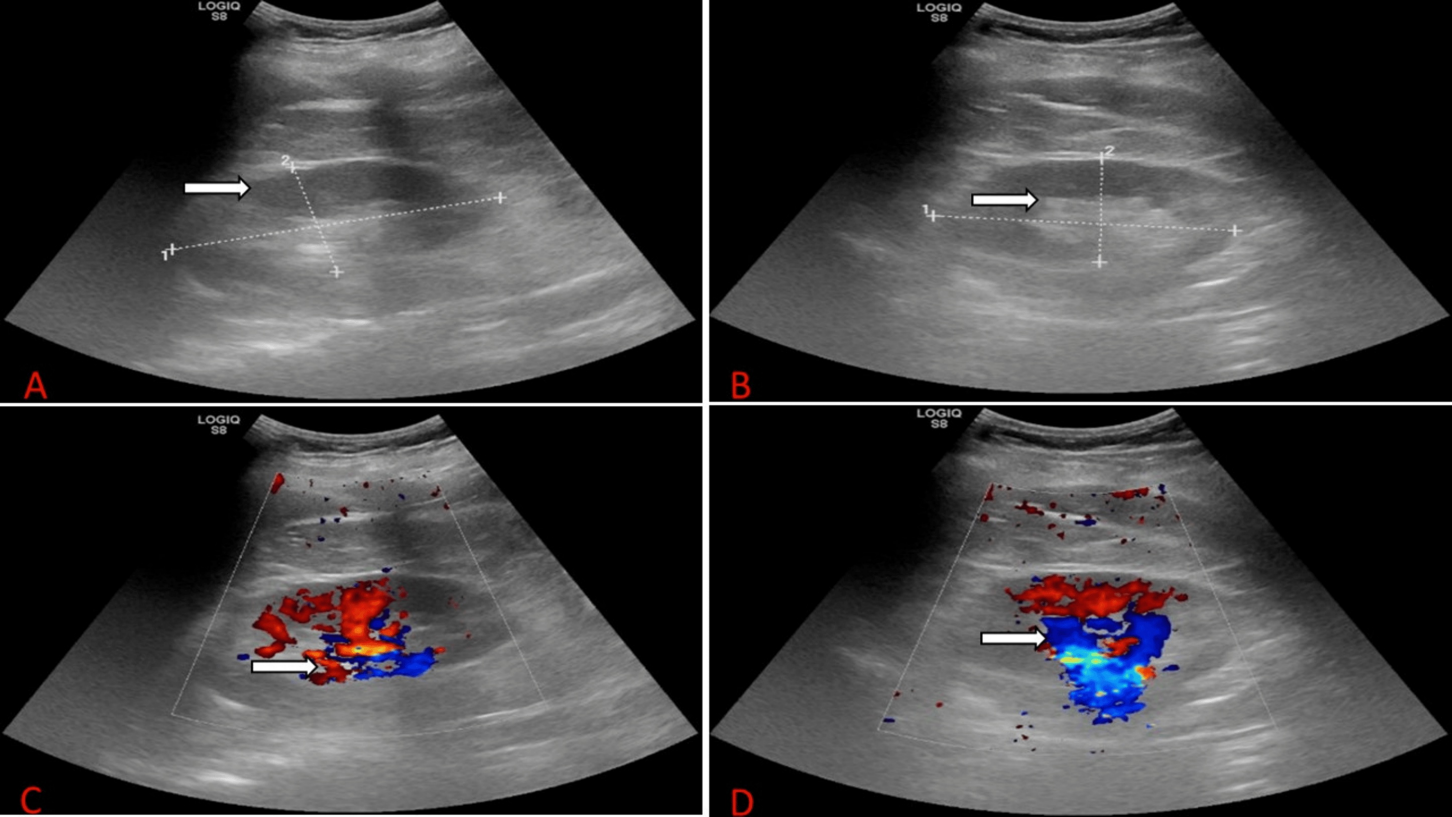
The kidney ultrasound done in 2024. A: The right kidney measuring 11.8 × 5.0 × 5.9 cm with mildly increased corticomedullary echogenicity (arrow) but normal parenchyma thickness and contour, no pelvicalyceal dilatation, no calculi, no cysts, and no solid masses. B: The left kidney measuring 10.0 × 4.8 × 5.0 cm with mildly increased corticomedullary echogenicity (arrow) but normal parenchyma thickness and contour, no pelvicalyceal dilatation, no calculi, no cysts, and no solid masses. C: Kidney ultrasound with Doppler images (arrow) of the right kidney. D: Kidney ultrasound with Doppler images (arrow) of the left kidney.

Kidney ultrasound done in 2024 is presented in Figure 1. It showed mildly increased echogenicity but normal parenchyma thickness and contour, no pelvicalyceal dilatation, no calculi, no cysts, and no solid masses in both kidneys. Findings were suggestive of medical renal disease.

Kidney biopsy

**Figure 2:**
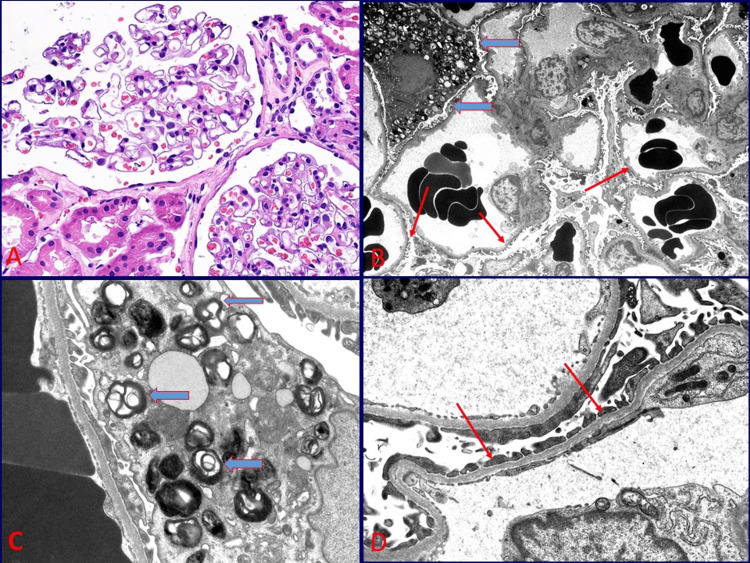
Kidney biopsy of the patient done in 2024. A: Renal cortical tissue with two glomeruli showing preserved capillary tufts and no significant changes (hematoxylin and eosin, ×200). B: Low magnification electron micrograph showing thin glomerular basement membranes (thin red arrows) that are otherwise preserved with foot processes and endothelial fenestrations. An enlarged podocyte/epithelial cells in the upper left corner contains numerous lipid inclusions (thick blue arrows) (×2,500). C: High magnification electron micrograph showing a podocyte containing concentrically layered lipid inclusions/myelin bodies within the lysosomes (thick blue arrow) (×12,000). D: High magnification electron micrograph showing thin glomerular basement membranes measuring under 200 nm (thin red arrows) (×12,000).

A kidney biopsy done in 2024 is presented in Figure 2. It showed minimal glomerular changes by light and immunofluorescence microscopy. However, electron microscopy demonstrated two abnormalities. The first finding was thin glomerular basement membranes, which could account for the persistent hematuria that was confirmed by a family history of hematuria as well as a pathologic variant of alpha-4 chain of type IV collagen. The second finding was evidence of lysosomal myelin inclusions within glomerular podocytes, resembling those seen in Fabry disease, which could contribute to a persistent subnephrotic proteinuric state.

The patient was referred to a dermatologist and an ophthalmologist for the workup of Fabry disease following his kidney biopsy results. Both eye and skin examinations were normal. Alpha-galactosidase level was normal. Based on clinical presentation and workup, the patient was diagnosed with thin glomerular basement membranes associated with the adult/late-onset variant of Fabry disease. The patient was started on Losartan 25 mg to manage moderate proteinuria (UPCR: 241 mg/g) and control blood pressure. The patient’s chronic kidney disease is currently stable, and he is under ongoing close monitoring in our clinic.

## Discussion

TBMN is a common disorder that results from mutations in genes encoding type IV collagen, leading to widespread and uniform thinning of the glomerular basement membrane [10]. This leads to microscopic hematuria, typically with normal kidney function and the absence of proteinuria [10]. Our patient had both personal and family history of hematuria as well as moderate proteinuria with UPCR of 241 mg/g. His workup confirmed a heterozygous inheritance pattern of alpha-4 chain of type IV collagen.

Fabry disease is an X-linked inborn error of the glycosphingolipid metabolic pathway that results from the accumulation of glycosphingolipids, especially globotriaosylceramide (Gb3), in different cells of the body, leading to a wide variety of clinical manifestations [11]. This non-specific and varied clinical presentation makes diagnosis challenging. Some of the earliest clinical presentations of Fabry disease encompass corneal and lenticular opacities, skin lesions, severe neuropathic or limb pain (acroparaesthesia), hypohidrosis, and gastrointestinal symptoms [12]. These presentations typically manifest in early childhood or young adulthood [12]. Late-onset/adult disease may have different clinical features, such as cardiac and renal presentation, which may further delay diagnosis [13].

In this case, we highlight the coexistence of thin glomerular basement membranes, an entity within the spectrum of Alport disease associated with the genetic defects of type IV collagen, along with a lysosomal lipid storage disease resembling Fabry disease. Such lesions can sometimes be masked by a pre-existing condition, as in this case, associated with hematuria, making diagnosis challenging. However, kidney biopsy played a crucial role in confirming the morphologic features of both entities. Secondary forms of accumulation of myeloid bodies (zebra bodies), which are sometimes observed in association with certain medications, could be considered in this setting but were excluded by the patient’s clinical history.

One of the common initial renal findings of Fabry disease is proteinuria, occurring in approximately 50% of untreated males by the age of 35 years [3]. The prevalence of proteinuria in males increases with age, reaching about 90% by the age of 50 years [3]. A significant proportion of patients with Fabry disease develop chronic kidney disease (CKD) and subsequently end-stage renal disease (ESRD) [3]. In a study by the National Institutes of Health that included 105 males with classic Fabry disease, all patients who survived to the age of 55 years developed ESRD [3]. Proteinuria and CKD are the typical presentations of Fabry disease, but our patient presented with hematuria. Although genetic testing indicated the presence of a type IV collagen-associated basement membrane disease, the biopsy findings identified two unrelated abnormalities, both of which could be associated with inherited defects causing disease. This case underscores the phenotypic heterogeneity of Fabry disease, particularly in adults, demonstrating an atypical presentation that can be easily overlooked without thorough investigation [14]. The patient’s presentation of hematuria could be due to the thin basement membrane, as the typical presentation of thin basement membrane disease is hematuria.

A diagnosis of Fabry disease typically requires genetic confirmation of mutation or reduced activity of α-galactosidase A enzyme activity, especially in males [15]. However, in rare cases, histopathological findings alone, such as the presence of myelin bodies (concentric lamellated lipid), inclusions of Gb3 in podocytes, endothelial, and tubular cells, may help in making a diagnosis, when other acquired causes of such accumulation are excluded [15]. Our patient had the pathologic variant of alpha-4 chain of type IV collagen, although the alpha-galactosidase level was normal.

## Conclusions

This is an unusual case presentation of two unrelated, possibly inherited genetic defects manifesting within the kidney tissue and detected mainly by electron microscopy. Hence, a kidney biopsy can be a valuable tool in identifying the characteristic histopathological features of clinically silent or asymptomatic disease, aiding in the diagnosis and management. These findings can potentially help with family counseling in the setting of inherited diseases.
